# Emerging Role of Epigenetic Alterations as Biomarkers and Novel Targets for Treatments in Pancreatic Ductal Adenocarcinoma

**DOI:** 10.3390/cancers14030546

**Published:** 2022-01-21

**Authors:** Marcus T. T. Roalsø, Øyvind H. Hald, Marina Alexeeva, Kjetil Søreide

**Affiliations:** 1 Department of Quality and Health Technology, University of Stavanger, 4036 Stavanger, Norway; marcus.t.roalso@uis.no; 2 HPB Unit, Department of Gastrointestinal Surgery, Stavanger University Hospital, 4068 Stavanger, Norway; marina.alexeeva@sus.no; 3 Gastrointestinal Translational Research Unit, Laboratory for Molecular Medicine, Stavanger University Hospital, 4068 Stavanger, Norway; 4 Department of Oncology, University Hospital of North Norway, 9038 Tromsø, Norway; oyvind.holsbo.hald@unn.no; 5 Department of Clinical Medicine, University of Bergen, 5020 Bergen, Norway

**Keywords:** pancreatic ductal adenocarcinoma, epigenetics, liquid biopsy, tumor microenvironment, immune therapy

## Abstract

**Simple Summary:**

Epigenetic alterations cause changes in gene expression without affecting the DNA sequence and are found to affect several molecular pathways in pancreatic tumors. Such changes are reversible, making them potential drug targets. Furthermore, epigenetic alterations occur early in the disease course and may thus be explored for early detection. Hence, a deeper understanding of epigenetics in pancreatic cancer may lead to improved diagnostics, treatments, and prognostication.

**Abstract:**

Pancreatic ductal adenocarcinoma (PDAC) is a lethal disease with limited treatment options. Emerging evidence shows that epigenetic alterations are present in PDAC. The changes are potentially reversible and therefore promising therapeutic targets. Epigenetic aberrations also influence the tumor microenvironment with the potential to modulate and possibly enhance immune-based treatments. Epigenetic marks can also serve as diagnostic screening tools, as epigenetic changes occur at early stages of the disease. Further, epigenetics can be used in prognostication. The field is evolving, and this review seeks to provide an updated overview of the emerging role of epigenetics in the diagnosis, treatment, and prognostication of PDAC.

## 1. Introduction

Pancreatic ductal adenocarcinoma (PDAC) remains a cancer with high mortality [1]. This is in large part due to late diagnosis impeding surgical therapy, as most patients present with locally advanced or metastatic disease. Important drivers of poor outcomes are found in the aggressive tumor biology, with lackluster response rates to many conventional drugs and propensity to early development of metastasis, despite the use of modern cytotoxic drug regimens (such as FOLFIRINOX). Though progress has been made in understanding the underlying disease mechanisms, several questions remain unanswered [2]. The main genetic mutations driving the disease, such as activation of KRAS, or inactivation of tumor suppressors TP53 and CDKN2A, have all proved difficult to amend with targeted therapies, thus limiting the current medical treatment options [3,4]. Therefore, the need for alternative treatment targets remains strong.

Epigenetic alterations have been linked to cancer for several decades. Increasingly, epigenetic mechanisms are appreciated as crucial drivers of the malignant phenotype [5,6] and several epigenetic changes are found in PDAC [7,8,9,10,11,12]. As the epigenome is involved in several aspects of tumorigenesis, epigenetic alterations may also serve as therapeutic targets. Nonetheless, the epigenetic field is in its relative infancy, with few drugs in clinical use for treating solid tumors [13].

Epigenetic alterations appear early during the disease course, making them candidates for potential diagnostic as well as prognostic biomarkers [14]. As PDAC is largely asymptomatic prior to advanced disease, early diagnosis is imperative to improve diagnosis. Thus, liquid biopsies from blood and pancreatic juices appear a promising addition in the diagnosis and treatment of PDAC.

While immunotherapy is increasingly used in solid organ cancer care, the effect is lacking in PDAC, as most patients do not respond to these novel therapies [15]. Since epigenetic processes also regulate the underlying immune cell functions resulting in an antitumoral response, combining immunotherapy and epigenetic therapies carries the potential to further benefit patient outcomes [9,16].

The aim of this review is therefore to explore the current understanding of epigenetics in PDAC and summarize recent advances in the field of epigenetic biomarkers and therapeutics, and its potential implication for immuno-oncology.

## 2. Materials and Methods

This is a narrative review based on a systematic literature search of the English literature, of PubMed/Medline indexed journals using key search terms including “epigenetics” AND “epigenetic alterations” AND “epigenetic changes” with “pancreatic cancer AND “pancreatic ductal adenocarcinoma”, focusing on recent authoritative reviews from the last 5 years (up to December 2021) and novel breakthroughs as reported in the literature.

## 3. Background

Epigenetic changes are heritable DNA modifications affecting gene expression [17]. They do not involve changes in the nucleotide sequence of DNA and are reversible, making them targets for tumor-directed therapies [18]. Epigenetic alterations are key in normal physiology and underpin how the transcriptome of the human body arises from an invariable genome. The epigenome can be influenced by external exposures, such as nutrition and life habits, and biological processes, such as ageing and inflammation [5]. Importantly, epigenetic changes may partake in carcinogenesis, as mutations of epigenetic modifiers are frequently seen in a variety of cancers.

The epigenetic changes occur through methylation of DNA, by histone modifications and through changes in chromatin structures. Epigenetic regulation and the corresponding changed chromatin states can activate oncogenes and silence tumor suppressor genes, prompting cancer cell proliferation, evasion of growth suppressors and resistance to apoptosis.

### 3.1. Epigenetic Changes in PDAC

**Figure 1:**
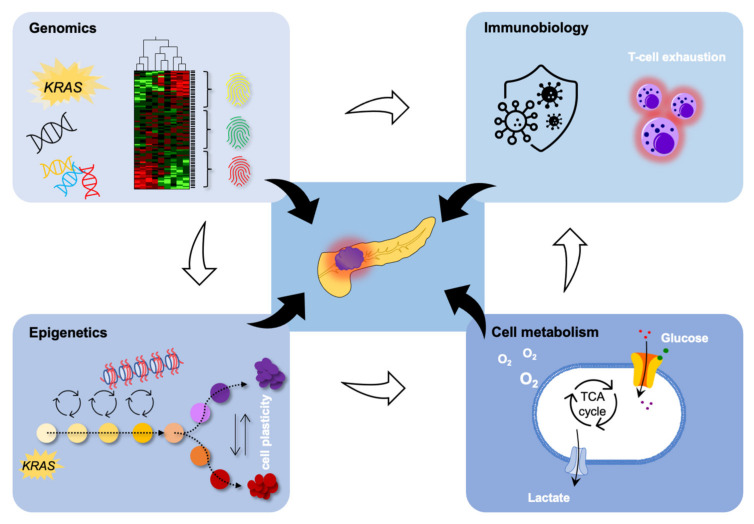
Epigenomics is strongly interrelated with genomics to affect cell metabolism and immune biology in PDAC. Please refer to the main body of text for details.

In PDAC, epigenetic changes are frequently found in genes partaking in oncogenic signaling, metabolic alterations, and the metastatic process [12,19], as depicted in Figure 1.

Deep whole-genome sequencing reveals that variations in chromosomal structures are important factors of DNA damage in pancreatic carcinogenesis, partially due to inactivation of chromatin modifiers, suggesting that epigenetic alterations influence tumor progression [20]. For instance, when studied in vitro and in vivo, primary human PDAC cells reprogrammed to reset their epigenetic profile demonstrated decreased tumorigenicity [21]. However, genetic mutations cannot predict the tumoral heterogeneity seen in PDAC with varying treatment responses [22]. Using PDAC xenografts in mice, subtyping using epigenetic, transcriptional, and stromal determinants suggests that genetic aberrations are responsible for the development of PDAC, while the responses to treatments are controlled at the epigenetic level [23].

PDAC is currently classified into several subgroups, ranging from 3 to 5 subtypes depending on the system used [24,25,26]. Two major molecular subtypes of PDAC have been found through transcriptome profiling, namely the classical and basal type [27]. The classical type has a better prognosis, demonstrating a clinical relevance. Studying the epigenomic landscape, the basal subtypes have altered methylation of effectors and inhibitors of the Wnt signaling pathways. Tumors of the classical type have hypomethylation with the corresponding overexpression of cholesterol transporter NCP1L1 [28]. In addition, basal tumors were found to have deregulation of several genes related to known oncogenic signaling networks, not confined to MYC, ErbB/EGFR, and TGFβ pathways.

The Wnt signaling pathway is a complex network connected to a wide range of biological processes, such as cell proliferation, differentiation, and migration, where mutation-induced activation of tumor suppressors promotes malignant disease [29]. Obesity is a well-known risk factor for PDAC, and patients with a high body mass index prior to diagnosis experience decreased survival [30]. Further, early metastasis is common in PDAC due to early epithelial-to-mesenchymal transition (EMT). One suggested mechanism for this is related to adipokines released from adipocytes. Adipokines induce EMT in pancreatic preneoplastic lesions via paracrine signaling activating the non-canonical Wnt signaling pathway receptor tyrosine kinase like orphan receptor 2 (ROR2) [31]. High ROR2 expression in PDAC is linked to a poor prognosis [32]. Targeting Wnt signaling may therefore also be an attractive cancer therapy model for PDAC.

Wnt signaling is under epigenetic control in cancers through hyperactivation of positive regulators or hypoactivation of negative regulators, modulated by various non-coding RNAs and chromatin modifiers [33]. Although genetic alterations are found in most cancers, there is a high degree of tissue specificity for mutations in individual components. The most frequent mutation in PDAC is in the Ring Finger Protein 43 (RNF43) gene, which causes inappropriate Wnt signaling and hyperplastic growth in a murine knockout model, due to a failure to downregulate Wnt receptors [34,35].

Epigenetic variation predicts the risk of tumor development and growth, and the DNA methylome in PDAC is significantly altered from healthy controls, with several protein coding genes and long non-coding genes being potential prognostic biomarkers [36,37]. In addition, long non-coding RNAs (lncRNAs) and microRNA expression profiles may help prognosticate patients [38,39]. For one, microRNA-192 is epigenetically downregulated in PDAC via promoter methylation, where higher expression caused better overall survival, together with in vitro data suggesting an inverse correlation with EMT markers and less favorable growth patterns [40]. Although no single epigenetic biomarker has been shown to provide strong diagnostic and prognostic value in PDAC for clinical use, a future panel detailing several epigenetic alterations might be of prognostic value [41].

One well-known risk factor for PDAC is chronic pancreatitis, which fits the already established general association between tumors and inflammation [42]. Epigenetic changes are linked to the early stages of oncogenesis in pancreatic cancer driven by inflammation [10,11]. The damage experienced by pancreatic epithelium during bouts of pancreatitis causes sustained transcriptional and epigenetic reprogramming, providing an epithelial memory, which is protective during future insults. This acinar ductal metaplasia is stabilized by *Kras* mutations, the main oncogenic driver mutation in PDAC, putting these clones under a strong positive selection during recurrent pancreatitis, facilitating tumorigenesis. Thus, targeting the epigenome at an early stage can possibly prevent cancerous disease in at-risk patients.

### 3.2. Tumor Microenvironment

The tumor microenvironment (TME) comprises a wide array of both cellular and non-cellular components, all of which are involved in tumor development, progression, and metastasis. These processes are in part driven by epigenetic mechanisms [43,44]. A histopathological hallmark of PDAC is stromal desmoplasia, with excessive deposition of extracellular matrix as a response to invasive cancer cells [45].

The chief contributor to desmoplasia in PDAC is pancreatic stellate cells (PSCs), which are in the minority in the healthy pancreas and usually appear in a quiescent state. Upon activation they grow in numbers and produce increasing amounts of ECM components, resulting in fibrosis [46]. However, other cells, such as resident fibroblasts and tumor-infiltrating mesenchymal stem cells (MSCs) partake in this process and make up the heterogenous cell population termed cancer-associated fibroblasts (CAFs). This fibrotic process also creates a hypoxic microenvironment and a barrier for immune cell influx and efficient drug delivery to the tumor cells [47,48]. With the addition of altered vascularization and infiltration of surrounding immune cells, the tumor mimics a wound with impaired healing, demonstrating that cancerous cells modulate the microenvironment to further growth [49,50].

Epigenetic changes can occur both by cell-based contact and through secreted factors [44]. DNA methylation of the *SOCS1* gene, a suppressor of cytokines and growth factors promoting cancerous growth, have been shown to be induced by PDAC cells, leading to increased tumor cell growth in vitro [51]. This is supported by clinical data showing an increased overall survival of 3 months in patients lacking *SOCS1* methylation.

Targeting oncogenic KRAS has been unsuccessful in PDAC, although progress has been made in other cancers [52]. In a study exploring whether the TME was the cause of therapy resistance in PDAC, *HDAC5* (Histone Deacetylase 5) was found to enable KRAS-independent growth by altering the TME myeloid cell composition, through the recruitment of tumor-associated macrophages (TAMs) via the HDAC5-CCL2 axis, with several potential drug targets [53]. In addition, by directly targeting TAMs in a murine PDAC model using the cytotoxic drug trabectedin, which induces caspase-8-dependent apoptosis in monocytes and macrophages, infiltrating T cells regained a favorable epigenetic profile and cytotoxic capabilities, indicating that TAMs influence the epigenetics of tumor infiltrating T cells [54].

The tumor in PDAC is characterized by a lack of T-cell infiltration due to an immunosuppressive TME and is largely refractory to immune-checkpoint inhibition (ICI) therapy [55]. Interestingly, the mutational load or the predicted number of neoantigens does not explain the variation in tumor infiltrating T cell numbers [56,57]. One reason for this seems to be that the epigenetic status in the tumor influences the immune environment, including in PDAC [58,59,60]. One of these tumor intrinsic epigenetic factors in PDAC is ubiquitin-specific protease 22 (USP22), which is suggested to control the immune TME through transcriptional regulation; thus, future inhibition of USP22 may turn the tumor sensitive to ICI [61]. A potent epigenetic regulator of immunotherapy response in PDAC is lysine demethylase 3A (KDM3A) [9]. This enzyme regulates the expression of epidermal growth factor receptor (EGFR). Cancer cells lacking KDM3A form tumors with infiltrating immune cells sensitive to immunotherapy. Therefore, when treating established tumors in mice with the EGFR inhibitor erlotinib, increased intratumoral T cells were found in a dose-dependent manner, displaying an altered TME and tumor cells sensitized to immunotherapy.

### 3.3. Metastasis

Metastatic disease in pancreatic cancer is invariably fatal and has limited treatment options. As PDAC progresses, tumor cell subclones emerge that have gained the capacity for spread, leading to distant metastasis. Understanding the molecular events underpinning the progression from primary tumor to metastatic lesions is likely essential to devise future curative treatment approaches. The genomic landscape of PDAC has been thoroughly mapped over the last decade, and several mutations affecting key genes driving the disease have been described. Interestingly, recurrent genetic events driving PDAC metastasis have not been identified [62]. When comparing the genetic alterations in metastases with that of primary tumors, it has been shown that identical PDAC driver mutations are shared by all subclones [63]. This finding implies that distinct epigenetic alterations may be underlying the metastatic phenotype.

Large-scale reprogramming of chromatin modifications during the evolution of PDAC metastasis was investigated [19] using both ChIP-seq, whole-genome bisulfite sequencing and immunohistochemistry-based approaches, demonstrating the disruption of H3K9, H4K20 and DNA methylation within large heterochromatin domains during the evolution of distant metastasis. A key finding in this study was the coupling of epigenetic alterations in distant metastatic cells to metabolic signaling, more specifically the pentose phosphate pathway (PPP). Pharmacologic inhibition of 6-phosphogluconate dehydrogenase (PGD, a rate limiting step of PPP, using antimetabolite 6AN led to a reversal of global chromatin changes, reduced the expression of overexpressed genes with malignant functions, and repressed tumorigenic properties in lung metastatic PDAC cells.

In a recent study [64], the chromatin structure of surgically resected tumors of patients with disease free survival >1 year was compared with patients with disease free survival <1 year; the latter group thus consisted of patients with early recurrence/metastatic relapse. Using ATAC sequencing, a technique used to probe for open chromatin by using an enzyme which cleaves DNA in open chromatin in combination with high resolution deep sequencing, they identified nearly 1100 differentially accessible chromatin peaks between these two groups. Further analyses showed that several of these regions contained transcription factor binding motifs, suggesting that changing the accessibility of transcription factor binding might serve as a mechanism promoting metastasis in PDAC. An in-depth analysis of two transcription factors (ZKSCAN1 and HNF1B) with binding motifs within differentially accessible chromatin loci showed a clear differential nuclear expression of these proteins in patients with regards to prognosis. Using a PDAC TMA cohort of 97 tumors, the authors demonstrated that high ZKSCAN1 expression corresponded to a poorer prognosis and high HNF1B was a feature of tumors in patients with longer survival. These findings corresponded nicely to the ATAC seq data which showed ZKSCAN1 motifs to be differentially open in recurrent patients and HNF1B motifs to differentially open in non-recurrent patients. The results from this study show that analyzing tumors for the expression of transcription factors associated with differentially expressed chromatin loci can serve as a novel prognostication tool in PDAC and predict early recurrence and metastasis.

The epigenetic eraser histone deacetylase 2 (HDAC2) has previously been shown to be connected to undifferentiated pancreatic cancer [65,66]. A more recent study explored the role of this enzyme with regards to PDAC metastasis [67]. In this paper, the authors demonstrated that HDAC2 controlled metastasis in a genetically engineered mouse model, where the metastatic burden in Hdac2 deficient mice was drastically reduced. Mechanistically, they showed that HDAC2 protected undifferentiated PDAC cells from the tumor suppressive functions of TGFβ signaling. Furthermore, HDAC2 was shown to maintain the expression of a genetic signature linked to undifferentiated PDAC. Collectively, the results from this study establish HDAC2 as an important epigenetic regulator of PDAC tumor biology and metastasis.

## 4. Epigenetic Biomarkers in PDAC

**Figure 2:**
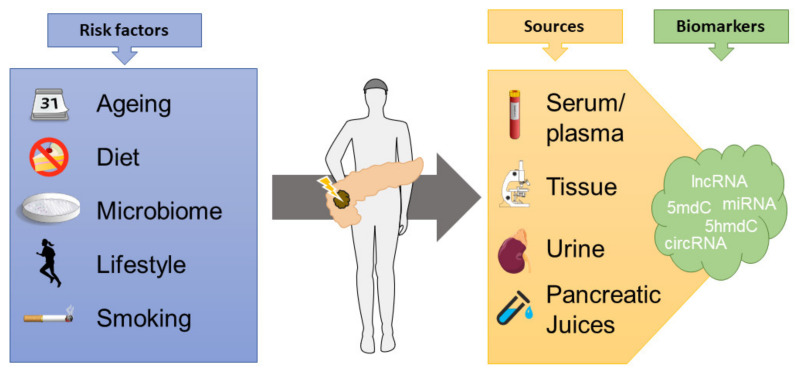
Several modifiable and non-modifiable factors influence the epigenome in cancers, which can then be explored to determine diagnostic and prognostic aspects, measuring epigenetic biomarkers from tissue specimens or bodily fluids (lncRNA = long non-coding RNAs, 5mdC/5hmdC = 5-methyl or 5-hydroxymethyldeoxycytidine, miRNA = microRNA, circRNA = circular RNA).

Epigenetic biomarkers have emerged as a promising tool for diagnostics and prognostication in cancerous disease, though none are currently in routine clinical use in PDAC (Figure 2). The DNA methylation profile changes during the early precursor stages of pancreatic cancer and serve as a driving force of tumor progression [68].

Several different types of biological materials, such as tissue samples from resected tumors or bodily fluids, can be used for validation of specific methylated DNA markers (MDM) as biomarkers for early-stage diagnosis [68]. One study utilized endoscopically obtained pancreatic juices from patients with biopsy-verified PDAC and healthy controls [69]. It showed that three groups of MDMs can be used as a biomarker, distinguishing patients with any stage of PDAC from controls with 83% sensitivity and identifying patients with stage I and II or intraductal papillary mucinous neoplasms (IPMN) with high-grade dysplasia with 80% sensitivity.

Biomarker validation in pancreatic juices demonstrated that the methylation level at the promoter regions of mucin genes can be used as diagnostic and prognostic biomarkers. Mucins are large membrane-bound glycoproteins playing a crucial role in carcinogenesis and tumor invasion in pancreatic tumors, and the gene expression is regulated by DNA methylation. MUC1, MUC2 and MUC4 gene methylation status measured by methylation-specific electrophoresis in pancreatic juices managed to distinguish cancerous precursor stages from PDAC [70]. Furthermore, methylation status of mucin genes analyzed by machine learning showed that higher hypomethylation level of MUC1 and MUC4 is associated with a poor prognosis [71].

Recent technical advances in sequencing allow the detection of epigenetic marks with high sensitivity in circulating cell-free DNA, opening the possibility for less invasive diagnostic assays [72]. For instance, the combination of two methods detecting 5-methylcytosine and 5-hydroxymethylcytosine improved the sensitivity for discrimination between stage I and II in patients with pancreatic cancer [73].

The circulating transcriptome contains diverse non-coding, stable and functional elements such as microRNAs (miRNAs), long non-coding RNAs (lncRNAs) and circular RNAs (circRNAs) [74]. Their resistance to RNase activity and readiness to be detected in the biological fluids of cancer patients showcase their potential as biomarkers in PDAC. Profiling miRNA expression can correlate to the stage of malignant pancreatic disease and hold potential as diagnostic and prognostic markers [75,76]. A retrospective study screening 2549 human miRNAs in patient sera showed that survival-related differentially expressed miRNAs were downregulated in patients with short-term survival compared to patients with long-term survival [77]. More specifically, the expression of hsa-miR-486-5p and hsa-miR-6126 were associated with long-term survival, and hsa-miR-3135b with short-term survival. Further, the ratio of mIR-3940-5p/miR-8069 detected in urine exosomes was elevated in early stages of PDAC [78]. When combined with the current biomarker gold standard for PDAC, Cancer Antigen 19-9 (CA 19-9), the ratio displayed a sensitivity of 93.0% and positive predictive value (PPV) 78.4%, increasing to a PPV of 100% when all markers were positive.

Several long non-coding RNAs (lncRNAs) have been shown to be involved in tumor progression in PDAC, and their detection in plasma or serum have been explored as potential biomarkers [79,80,81]. The Hox transcript antisense RNA (HOTAIR) lncRNA and hexokinase-2 (HK2) expression levels are elevated in the serum of patients with PDAC compared to healthy controls [82]. The group of PDAC patients with high expression levels of HOTAIR and HK2 demonstrated significantly lower overall survival rates compared with patients with low expression levels. Further, lncRNA LINC01111 acts as a tumor suppressor, with low expression levels associated with a poor prognosis [83].

Circular RNAs (circRNAs), that is non-coding RNAs in the form of covalently closed loops resulting from back-splicing of pre-mRNA transcripts, may have a clinical relevance in PDAC as biomarkers due to their influence on the transcriptome [84]. The circRNA circBFAR is involved in PDAC tumor progression causing proliferation and invasiveness via the cBFAR/miR-34b-5p/MET axis. CircBFAR acts as molecular sponge for miR-34b-5p, leading to upregulation of mesenchymal–epithelial transition factor (MET), causing tumor progression in PDAC. circBFAR was overexpressed in 208 patients with PDAC with a corresponding poor prognosis [85].

Various types of RNAs can be found in extracellular vesicles (EV) in blood, with messenger RNA (mRNA), circRNA, and lncRNA collectively termed long RNA (exLRs). EV-associated exLRs exhibit different profiles in patients with PDAC and healthy controls and is a potential diagnostic tool [86]. In a case-control study, a diagnostic signature comprising eight EV-associated exLRs in the plasma of PDAC patients had a high sensitivity identifying stage I and stage II PDAC. This suggests that exLR profiles may detect PDAC at an early resectable stage, and, importantly, can detect PDAC in patients without elevated levels of the tumor marker CA 19-9, distinguished from healthy controls [87].

## 5. Epigenetic Therapeutic Options

Several epigenetic modulators have undergone clinical testing, though few have entered routine clinical use [88]. No epigenetic drugs are currently in use in treating pancreatic cancer (Table 1). The first generation of epigenetic drugs date back to the 1960s and were unspecific and troubled with side effects. The last two decades has seen nine drugs gaining FDA approval, however they are mostly limited to hematological malignancies [18]. The new generation of epigenetic modifier drugs are more selective, and many target proteins mutated or translocated in cancer [89].

Consequently, several compounds have been evaluated and clinical trials have included patients with PDAC. Curcumin, a p300 histone acetyltransferase inhibitor, and histone deacetylase (HDAC) inhibitor vorinostat, have demonstrated favorable responses in select patients, however no meaningful conclusions can be drawn based on limited experience [88]. Moreover, vorinostat and the cytotoxic drug capecitabine, an oral fluoropyrimidine, have been used in combination in a neoadjuvant chemoradiation regimen, though this was limited to a phase I trial [90].

Although monotherapies using epigenetic drugs are without benefit, targeting epigenetic modifications can alter susceptibility of PDAC towards standard of care chemotherapies. In addition, drug regimens combining drugs targeting different chromatin regulators are a promising avenue of research. Further, it has been shown that epigenetic therapies alter several immuno-oncological mechanisms, which have sparked interest in combining epigenetic therapies with immunotherapy [91].

Improved gemcitabine delivery was seen in preclinical studies through inhibition of Hedgehog (Hh) signaling [92]. Clinical trials ensued in several cancerous diseases, however these failed and did not enter phase III trials. Nonetheless, preclinical studies with epigenetic targeting of bromo and extra C-terminal (BET) bromodomain proteins, which regulate the downstream transcriptional output of Hh signaling, showed promising effects in vitro, showcasing potential synergistic treatment options [93]. BET proteins are considered important players in PDAC development and are an active area of research [94].

The strategy to induce subtype switching in PDAC has been further explored based on the transcription factor GATA-binding factor 6 (GATA6) role as a regulator of classical PDAC subtype identity [28]. Depletion of GATA6 enforces a basal-like state in PDAC [95]. The histone methyltransferase enhancer of zeste homolog 2 (EZH2) is a transcriptional regulator of GATA6 in PDAC and blocking EZH2 reinstated GATA6 and induced gene signatures seen in the classical PDAC subtype, which confers a survival benefit [96]. Therefore, EZH2-GATA6 axis is a potential target in future PDAC treatment. The EZH2 inhibitor tazemetostat is approved by the FDA for use in the treatment of advanced epithelioid sarcoma and the drug is further explored in a phase II trial in combination with ICI in other solid tumors including PDAC (NCT04705818).

In addition, epigenetic inactivation of GATA6 promotes the differentiation of aggressive squamous-like subtypes in PDAC [97]. This epigenetic deregulation was shown using genome-wide epigenetic mapping of modifications 5-methylcytosine and 5-hydroxymethylcytosine (5hmC). These transcriptional subtypes show a greater loss of 5hmC due to reduced expression of 5-methylcytosine hydroxylase TET2. Concurrently, SMAD4 back TET2 levels in the classical PDAC subtype, and loss of SMAD4 expression displayed reduced 5hmC and GATA6 resulting in a more squamous-like tumor. In one study, the development of squamous-like subtypes in PDAC were reversible in vivo in a murine model combining the antidiabetic drug metformin and ascorbic acid/vitamin C, restoring 5hmC and GATA6 levels, which in turn reverted the squamous-like phenotype. If proven to have clinical relevance, there may be mechanisms by which therapeutic resistance can be overcome and with it the possibility of increased survival.

Epigenetic regulation is vital for normal cell function, requiring high specificity to reduce potential side effects, which first hindered progress in the field [98]. Although side effects are manageable, the addition of epigenetic drugs to established treatments often leads to unwanted effects. In a phase II trial exploring CI-994, an oral HDAC inhibitor combined with gemcitabine was compared to placebo and gemcitabine; patients in the treatment arm experienced significantly more treatment-related NCT03250273 events, such as neutropenia, thrombocytopenia, and fatigue [99]. Similar drug-related adverse events were found in a phase II trial of the dual Polo-like kinase/BRD4 bromodomain BET inhibitor BI 2536 in chemo-näive unresectable PDAC patients [100].

### 5.1. Tumor Microenvironment

Depletion of the desmoplastic stroma seeks to improve delivery and response towards chemotherapeutic drugs. A major goal of antifibrotic therapy is to target CAFs, which produce ECM components [101]. Initial studies of now standard of care chemotherapeutic drugs nab-paclitaxel and gemcitabine demonstrated significant antitumoral effects, decreasing CAF content prior to surgery [102]. However, immunohistochemistry surveying secreted protein acidic and rich in cysteine (SPARC), a glycoprotein constituent of the extracellular matrix that binds albumin and is over-expressed in PDAC, both in the tumor and surrounding stroma, and did not correlate with overall survival.

There is conflicting evidence regarding epigenetic manipulation of the TME. The TME contains a heterogenous cell compartment with varying responses to epigenetic treatments [103]. For instance, BET protein inhibition has increased efficacy when combined with HDAC inhibitors in PDAC, displaying reduced MYC activity and inflammation [104]. At the same time, HDAC inhibition has been shown to increase secretion of cytokines inducing a tumor supportive phenotype in pancreatic cancer associated fibroblasts [105]. Further, blocking DNA methylation using the DNMT inhibitor 5-azacytidine reduces PDAC progression [106]. Contrary to this, depleting DNMT increased the production of hyaluronic acid, using the same drug or DNMT knockdown by small interfering RNA, consequently promoting PDAC progression [107]. Transcriptomic and DNA methylomic analysis of epithelial cells from normal pancreata and PDAC revealed a subgroup characterized by hypomethylation of repetitive elements, which in turn activates an interferon-linked transcriptional program [108]. The low-methylation tumors were more aggressive compared to high-methylation tumors, which conserved cell-of-origin traits in a higher manner, suggesting cell-of-origin and epigenetics influence tumor heterogeneity. Therefore, it appears that optimal epigenetic treatments imply an act of balancing, possibly using multiple drugs targeting different epigenetic regulators, to obtain the best anti-tumoral efficacy in combination with cytotoxic drug regiments.

### 5.2. Immune Therapy

Infiltrating immune cells, especially T cells, carry the potential to eradicate a tumor, a capability which is currently utilized in treating a variety of cancers [109]. By targeting cellular check points in T cell priming and activation, the immune tolerant state is bypassed, reenergizing T cell reactivity towards tumor antigens. Currently this is achieved using antibodies targeting receptors PD-1 and CTLA-4 present on T cells, and their respective ligands PD-L1 and CD80 or CD86, expressed on tumor and antigen presenting cells (APCs). However, there are several other known checkpoints, and research is ongoing in combining them for increased efficacy [110].

Several prerequisites are needed for immune checkpoint inhibition (ICI) to work. Tumor-specific immune cells must be present in the TME, and these resident cells must be in a permissive state, which otherwise can turn off potential immune responses, a phenotype which is under epigenetic control [111,112]. Further, the tumor and immune cells must be reliant on the targeted mechanisms of immune escape, such as the PD-1-PD-L1 axis. This is often defined as immune ‘cold’ vs. immune ‘hot’ tumors [113].

Epigenetic therapies hold promise to enhance ICI therapies, especially in diseases like PDAC where combinatorial drug regiments are needed to overcome resistance to current immunotherapies. This can be achieved through modulating the TME, reprogramming T cell exhaustion, and more, which is reviewed in full elsewhere [114].

Checkpoint inhibition has been unsuccessful in PDAC, although patients with mismatch repair deficient tumors have seen favorable results [115]. To overcome therapy resistance in a murine model, epigenetic therapy priming using the DNA hypomethylating agent decitabine proved successful in modulating the TME [16]. There was an elevated number of tumor-infiltrating T cells, TAMs, and cytokine signaling, and increased antigen presentation in tumor cells, leading to an increased response to ICI therapy with significantly prolonged survival.

Transposable elements and tumor-associated antigens are potentially highly immunogenic. These are upregulated during the transition from a premalignant state to malignancy in a murine PDAC model, which coincides with a downregulation of antigen presentation and T cell recruitment, signifying immune evasion of tumor [116]. By treating mice with the DNA methyltransferase inhibitor 5-azacytidine, tumor regression was observed in immunocompetent mice, indicating that the immune system controlled tumor growth, with the epigenetic treatment enhancing tumor immunogenicity.

Myeloid-derived suppressor cells (MDSCs) are recruited into tumors, where they inhibit T cell infiltration and activation. This is detrimental to T cell anti-tumor activity, rendering ICI therapy less effective [117]. The histone deacetylase inhibitor entinostat, together with anti-PD-1 and anti-CTLA-4 therapy, significantly improved the survival in a murine metastatic pancreatic cancer model [118]. This happened through decreased MDSC-mediated suppression in the TME and an increase of activated CD8+ T cells, leading to a concurrent change in immune-related pathways as per gene-expression profiling. These results led to a phase II clinical trial awaiting results (NCT03250273).

## 6. Future Directions

Epigenetic changes are increasingly understood as a potential diagnostic and therapeutic target in PDAC. Although no treatments are in clinical use, several drugs are undergoing preclinical evaluation, at this point often in combination with other therapies due to synergistic effects. Even though PDAC up to now has been resistant to immune therapies, epigenetics may increase the efficacy of ICI, with the promise to help treat patient subpopulations, as seen in other malignancies. Understanding both the epigenetic alterations, and the TME, is therefore likely key to improving the treatment options in PDAC.

## Figures and Tables

**Table 1:** List of current and prior trials with epigenetic therapies in PDAC.

Drug(s)	Combination Agent(s)	Phase of Study	Status	NCT Number
Panobinostat Vorinostat	Various antineoplastic drugs	Phase 1	Recruiting	NCT03878524
Tazemetostat	Durvalumab	Phase 2	Recruiting	NCT04705818
Romidepsin Azacitidine	Gemcitabine Durvalumab Lenalidomide nab-Paclitaxel	Phase 1/2	Recruiting	NCT04257448
Azacitidine	Chemotherapy after progression	Phase 2	Active, not recruiting	NCT01845805
Vorinostat	Gemcitabine Sorafenib	Phase 1	Active, not recruiting	NCT02349867
Azacitidine	Pembrolizumab	Phase 2	Active, not recruiting	NCT03264404
Entinostat	Nivolumab	Phase 2	Completed	NCT03250273
Decitabine	Tetrahydrouridine	Phase 1	Completed	NCT02847000
Entinostat		Phase 1	Completed	NCT00020579
Vorinostat	Capecitabine	Phase 1	Completed	NCT00983268
Azacitidine	nab-Paclitaxel Carboplatin	Phase 1	Completed	NCT01478685
Vorinostat	NPI-0052 (marizomib)	Phase 1	Completed	NCT00667082
Panobinostat	Bortezomib	Phase 2	Terminated (funding not available)	NCT01056601
Entinostat	FOLFOX regimen	Phase 1	Withdrawn (lack of funding)	NCT03760614
Entinostat Molibresib		Phase 1	Withdrawn (protocol moved to disapproved)	NCT03925428
Vorinostat		Phase 1/2	Terminated (slow accrual)	NCT00831493
Vorinostat	5-FU	Phase 1/2	Terminated (funding withdrawn)	NCT00948688
Azatacidine	Gemcitabine	Phase 1	Terminated (miscellaneous reasons)	NCT01167816
	Epigenetic targets			
Azacitidine	Hypomethylates DNA by inhibition of DNA methyltransferase (DNMTi), halting cell division.			
Decitabane				
Tazemetostat	Lysine histone methyltransferase inhibitor (HMTi), selective inhibition of EZH2.			
Molibresib	Molibresib is a bromodomain and extra-terminal motif inhibitor (BETi), downregulating transcription of oncogenes.			
Vorinostat	Histone deacetylase inhibitors (HDACi) that induce growth arrest, differentiation, autophagy, and apoptosis in tumor cells.			
Entinostat				
Panobinostat				
Romidepsin				

Legend: Table is based on Roalsø et al. [14] and reproduced with permission from Springer © 2021.
